# Concurrent use of palbociclib and radiation therapy: single-centre experience and review of the literature

**DOI:** 10.1038/s41416-020-0957-9

**Published:** 2020-06-29

**Authors:** Arnaud Beddok, Hao Ping Xu, Alexandre Arsène Henry, Baptiste Porte, Alain Fourquet, Paul Cottu, Youlia Kirova

**Affiliations:** 1 Department of Radiation Oncology, Curie Institute, Paris, France; 2 Department of Radiation Oncology, Shanghai Jiao Tong University, Shanghai, China; 3 Department of Medical Oncology, Curie Institute, Paris, France; 4 Versailles University, Saint-Quentin-En-Yvelines, France

**Keywords:** Breast cancer, Breast cancer

## Abstract

Palbociclib in combination with endocrine therapy increases progression-free survival in patients with ER-positive, HER2-negative advanced breast cancer (BC). In this study, we retrospectively evaluated safety in the first patient treated with concurrent use of palbociclib and radiation therapy (RT) in the Curie Institute. Between April 2017 and August 2019, 30 women with metastatic BC received locoregional and/or symptomatic irradiation at a metastatic site concurrently with palbociclib. The most common acute toxicities were radiodermatitis and neutropenia. Palbociclib had to be discontinued during RT in three locally treated patients who developed grade 3 radiodermatitis and febrile neutropenia, grade 2 dysphagia and metastatic disease progression, respectively. After a follow-up of at least 6 months, none of the patients had late toxicity. Concomitant administration of palbociclib with RT was reasonably well tolerated in our series of 30 patients. More prospective data with longer follow-up are needed to confirm these results.

## Background

In the recent PALOMA-2 and PALOMA-3 clinical trials, addition of palbociclib, a CDK4/6 inhibitor, to standard endocrine therapy significantly improved outcomes in treatment of ER-positive, HER2-negative advanced breast cancer (BC).^1,2^ However, the risks associated with concomitant combination of palbociclib and radiation therapy (RT) are unknown, and the majority of radiation and medical oncologists prefer to suspend palbociclib during RT. The objective of this study was to retrospectively assess toxicity in a large number of patients treated with a combination of palbociclib and RT (Palbo-RT).

## Methods

We retrospectively analysed the data of 30 patients with de novo metastatic BC treated with palbociclib, and received irradiation to at least one site at Curie Institute between April 2017 and August 2019. Patients received LR irradiation if, after several months of palbociclib, the staging assessment showed at least a LR-partial response and stable metastatic disease. Palliative RT to a metastatic site was used to reduce pain when systemic analgesic treatment was not sufficient, or in the presence of metastatic epidural spinal cord compression.

In the case of locoregional irradiation, the choice of target volumes and doses was in accordance with the Institut Curie guidelines. Intensity-modulated radiation therapy (IMRT) was used for all LR treatments. For metastatic irradiation, the CTV usually included the macroscopic tumour volume with a variable margin. In this setting, a conformational three-dimensional technique was mostly used with prescribed doses of 20 Gy in five fractions (*n* = 13), 30 Gy in ten fractions (*n* = 10) and 8 Gy in one fraction (*n* = 3). The brain metastasis was treated stereotactically (one fraction of 18 Gy).

Palbociclib was initially administered at a daily dose of 125 mg (D), from D1 to D21 in combination with endocrine therapy: letrozole (with or without LHRH agonist) or fulvestrant, every 28 days. The palbociclib dose was reduced in the event of haematologic toxicity. For all included patients, RT was initiated randomly: either on the first day of the 3-week cycle, in a cycle already started, or even between cycles.

Follow-up was determined from the last day of RT until death or last examination. During RT, each patient was reviewed by a radiation oncologist each week to assess and treat any acute toxicities. Acute, like late toxicities, were scored by using Common Terminology Criteria for Adverse Events Version 5.0. For patients with evidence of local–regional recurrence or distant metastasis, additional examinations or imaging modalities were performed to confirm or exclude disease progression at the treating physician’s discretion.

## Results

The baseline characteristics of the 30 consecutive patients are summarised in Supplementary Information Table 1. A total of 35 sites were irradiated, including 9 (26%) LR sites, 17 (49%) sites in the spine, 7 (20%) sites in the peripheral skeleton, one in the brain (treated with stereotactic RT) and one choroidal metastasis. Table 1 summarises the characteristics of the nine locoregional irradiations.

**Table 1:** Characteristics of the nine locoregional irradiations.

Patient	Sites	CTVcc	PTVcc	Dose	Technique	Grade ≥ 2 acute toxicity	Palbociclib suspension due to toxicity
1	Left thoracic wall + left L1–L4 and IP	223	392	50 Gy (2 Gy/f)	Tomo	0	0
2	Right thoracic wall + right L2–L4, IP, and IMN	362	669	50 Gy (2 Gy/f)	VMAT	Neutropenia	0
3	Left breast + left L1–L4 and IP	566	820	50 Gy (2 Gy/f)	Tomo	0	0
4	Right breast + right L1–L4 and IP SIB	1082	1285	50.4 Gy (1.8 Gy/f) SIB: up to 64.4 Gy (2.3 Gy/f)	Tomo	0	0
5	Right thoracic wall + right L1–L4 and IP	395	725	50 Gy (2 Gy/f)	Tomo	0	0
6	Right thoracic wall + right L1–L4 and IP	511	805	50 Gy (2 Gy/f)	Tomo	0	0
7	Left thoracic wall + left L2–L4 and IP	533	778	50 Gy (2 Gy/f)	Tomo	0	0
8	Left breast + left L2–L4 and IP SIB	1355	1607	50.4 Gy (1.8 Gy/f) SIB: up to 64.4 Gy (2.3 Gy/f)	VMAT	Dermatitis, neutropenia and dysphagia	1
9	Bilateral thoracic walls + bilateral L1–L4 and IP	1019	1607	50 Gy (2 Gy/f)	Tomo	Dermatitis, neutropenia and pain	1

*L1–L4* axillary level 1–3 and supraclavicular region (level 4), *IP* interpectoral (Rotter) nodes, *IMN* internal mammary nodes, *SIB* simultaneous integrated boost, *2* *Gy/f* 2 Gy per fraction, *Tomo* tomotherapy, *VMAT* volumetric modulated arc therapy.

Treatment delivery compliance data are summarised in Supplementary Information Table 2. In most patients, palbociclib was administered at a daily dose of 125 mg (D), from D1 to D21 in combination with either fulvestrant 500 mg (9/30 patients) or letrozole 2.5 mg (21/30 patients) with or without LHRH agonist every 28 days. Mean overall treatment time of RT was 41 days (range, 35–53 days) for LR irradiation and 8.3 days (range, 1–14 days) for metastatic irradiation. The mean duration of concomitant administration of palbociclib and RT was 8.8 days (range, 1–24 days). It is interesting to note that the average half-life of palbociclib is 26 h. In three patients (LR treatment), palbociclib had to be discontinued during RT (due to toxicity in two cases, and metastatic disease progression in one case).

**Table 2:** Comparison of cohorts of patients concomitantly receiving CDK4/6 inhibitor and radiation therapy.

	CDK4/6 inhibitor	Patients	Number of irradiated sites	Location metastasis	LR	RT suspension required	CDKi suspension required	Grade 2 non-haematologic toxicities	Grade ≥ 2 haematologic toxicities
Hans et al.^3^ (6)	Palbociclib	5	5	Bone (4), visceral (1)	0	0	0	0	5 (100%)
Meattini et al.^4^ (11)	Ribociclib	5	8	Bone (5) and visceral (3)	0	0	2 (40%)	2 (25%)	1 (20%)
Kawamoto et al.^8^ (7)	Palbociclib	1	1	Bone (1)	0	1 (100%)	1 (100%)	1 (100%)	na
Kalash et al.^9^ (8)	Palbociclib	3	3	Lung (3)	0	3	3 (100%)	3 (100%)	na
Chowdhary et al.^5^ (12)	Palbociclib	16	23	Bone (18), brain (4) and visceral (1)	0	0	0	0	na
Messer et al.^10^ (15)	Palbociclib	1	1	0	1 (L4)	1 (100%)	1 (100%)	1 (100%)	na
Figura et al.^6^ (13)	Palbociclib and abemaciclib	15	42	Brain (42)	0	0	0	2 (5%)	na
Ippolito et al.^7^ (14)	Palbociclib and ribociclib	16	24	Bone (23)	1 (IMC)	2 (12%)	1 (6%)	1 (4%)	31%
Our study (2019)	Palbociclib	30	35	Bone (24) and brain (2)	9	2 (6%)	3 (10%)	3 (8%)	8 (26%)

*LR* locoregional, *IMC* intermammary chain, *CDKi* CDK inhibitor.

The acute toxicities most commonly observed during RT in our cohort were dermatitis and neutropenia. Detailed results are shown in Supplementary Information Table 3. Thirteen patients (43%) experienced grade ≥2 acute toxicities (nine had grade ≥2 neutropenia, two had grade ≥2 dermatitis, one had grade 2 dysphagia and one had grade 3 pain). Palbociclib had to be discontinued during RT due to toxicity in two patients (patients 8 and 9) with de novo metastatic BC receiving locoregional breast and lymph node RT (2/30): one patient developed grade 3 radiodermatitis and febrile neutropenia, while the other patient developed grade 2 dysphagia (loss of 4 kg in 1 month). Both patients had a particularly large planning target volume (PTV, ~1607 cc), whereas the mean PTV for locoregional irradiation was 965 cc (392–1607 cc). One of the patients receiving pelvic irradiation experienced grade 1 colitis, while no cases of cystitis or proctitis were observed. With a median follow-up of 17 months (6–31 months, SD ± 8) after completion of radiation therapy, no patient had experienced any late toxicity.

## Discussion

The clinical effects of the combination of palbociclib and RT are unknown. In 2017, Hans et al., in a short letter, reported the very preliminary results in five patients treated with this combination.^3^ RT was indicated in every case because of the symptoms of the disease: pain and/or compression. In four cases, external beam RT was administered for bone metastases to a total dose of 20 Gy in five fractions, whereas the fifth patient underwent stereotactic body RT for liver metastases to a total dose of 60 Gy in ten fractions. The authors did not observe any increased toxicity, particularly haematologic toxicity. The results of other retrospective studies that evaluated the combination of a CDK4/6 inhibitor and RT are summarised in Table 2. They all showed that grade ≥2 non-haematologic toxicities were rare and grade ≥2 haematologic toxicities did not appear to be increased compared with patients receiving CDK4/6 inhibitor alone.^4–7^ Conversely, several studies with a very small number of patients reported severe acute non-haematologic toxicities when a CDK4/6 inhibitor was administered concomitantly with RT.^8–10^ For instance, Kawamoto et al. observed severe acute radiation-induced enterocolitis after combined palbociclib and palliative RT in sacral metastasis.^8^ Kalash et al. also reported enhanced pulmonary fibrosis in three patients with metastatic breast cancer treated with palbociclib, letrozole and RT concurrently.^9^ In our cohort, no grade ≥2 toxicities other than haematologic toxicities were observed in the context of metastatic irradiation. Contrary to Kawamoto’s report, no patient with pelvic irradiation experienced cystitis or proctitis, and only one patient experienced grade 1 colitis, easily treated with symptomatic treatment.^8^ Haematologic toxicities were comparable to previous reports. It is interesting to note that the irradiated sites included only two sites in the pelvis, which is where major haematologic side effects from RT arise (main bone marrow site). The lack of increased significant haematologic toxicity in our study could be due to the site receiving RT.

One of the strengths of our study was to include nine patients (30%) with de novo metastatic breast carcinoma, who were at least stable after 6 months of palbociclib and received LR irradiation. Palbociclib had to be discontinued during RT in two of them because of grade 3 dermatitis and pain, and grade 2 oesophagitis, respectively. However, in both cases (patients 8 and 9), the PTV was particularly large compared with other LR irradiation volumes. It is therefore difficult to conclude that the palbo-RT combination was responsible for these toxicities.

This study, with the largest number of patients published to date, including LR irradiation, showed for the first time the acceptable safety profile of the combination of palbociclib and RT, with no unexpected or limiting adverse events. Given this experience, palbociclib should not be discontinued during radiation therapy. These results must be confirmed in a prospective study based on a larger number of patients.

## Supplementary information

Supplementary data

## Data Availability

The data that support the findings of this study are available from the corresponding author upon reasonable request.
