# Supplementary material for: Concurrent use of palbociclib and radiation therapy: single-centre experience and review of the literature
Source: Br J Cancer. 2020 Jun 29;123(6):905–8. doi: 10.1038/s41416-020-0957-9 (PMC7493877; doi:10.1038/s41416-020-0957-9)
Supplement: Supplementary file 1 — Supplementary data [file 41416_2020_957_MOESM1_ESM.docx]

Supplementary Material Table 1 : Baseline characteristics of the 30 patients

| Characteristics | Nb | % |
| --- | --- | --- |
| Number of patients | 30 |  |
|  |  |  |
| Median age (y,range) | 66 (35 - 86) |  |
|  |  |  |
| Pathology (WHO classification) |  |  |
| Invasive ductal carcinoma | 24 | 80 |
| Invasive lobular carcinoma | 6 | 20 |
|  |  |  |
| Immunochemistry |  |  |
| HER 2 expression |  |  |
| negative | 29 | 97 |
| positive | 1 | 3 |
| ER-positive | 30 | 100 |
| Grade |  |  |
| 1 | 3 | 10 |
| 2 | 16 | 53 |
| 3 | 6 | 20 |
| na | 5 | 17 |
| KI 67 |  |  |
| 0 – 10% | 3 | 10 |
| 10 – 20% | 6 | 20 |
| > 20% | 12 | 40 |
| na | 9 | 30 |
|  |  |  |
| Number of irradiated sites | 35 |  |
|  |  |  |
| Surgery before irradiation (in the irradiated site) |  |  |
| Total | 13 | 43 |
| Locoregional | 8 | 26 |
| Metastatic | 5 | 17 |
|  |  |  |
| Irradiated sites |  |  |
| Locoregional | 9 | 26 |
| Cervical Spine | 3 | 9 |
| Thoracic Spine | 7 | 20 |
| Lumbosacral Spine | 7 | 20 |
| Peripheral Skeleton | 5 | 15 |
| Pelvis Skeleton | 2 | 5 |
| Brain | 1 | 2.5 |
| Choroidal metastasis | 1 | 2.5 |
|  |  |  |
| Radiation therapy technique |  |  |
| 3D conformational | 24 | 69 |
| IMRT (VMAT or Tomotherapy) | 10 | 29 |
| Stereotactic Beam radiation therapy | 1 | 2 |
|  |  |  |

IMRT: intensity modulated radiation therapy, VMAT: volumetric modulated arc therapy

Supplementary Material Table 2 : Treatment delivery compliance

|  | Nb | % |
| --- | --- | --- |
| Overall treatment time (Palbociclib, months) | 12.4 (2 – 25) |  |
|  |  |  |
| Dose of Palbociclib |  |  |
| 125 mg | 27 | 90 |
| 100 mg | 1 | 3 |
| 50 mg | 2 | 7 |
|  |  |  |
| Concomitant hormonotherapy |  |  |
| Fulvestrant | 9 | 30 |
| Letrozole | 13 | 43 |
| Letrozole + LHRH | 8 | 27 |
|  |  |  |
| Number of lines of treatment before Palbociclib |  |  |
| 0 | 21 | 70 |
| 1 | 6 | 20 |
| > 1 | 3 | 10 |
|  |  |  |
| Overall treatment time (RT, days) |  |  |
| Locoregional | 41 (35 – 53) |  |
| Metastatic | 8.3 (1 – 14) |  |
|  |  |  |
| Time of concomitant Palbociclib and RT (days) | 8.8 (1 – 24) |  |
| Locoregional | 17 (5 – 24) |  |
| Metastatic | 5.8 (1 – 10) |  |
|  |  |  |

Supplementary Material Table 3: Incidence of major acute toxicities for the 30 patients (35 irradiated sites)

| Toxicities |  | Number of sites by toxicity grade (%) | | |  |
| --- | --- | --- | --- | --- | --- |
|  |  | Grade 0 | Grade 1 | Grade 2 | Grade 3 |
|  |  |  |  |  |  |
| Dermatitis | Total | 23 (66%) | 10 (28%) | 1 (3%) | 1 (3%) |
|  | Locoregional | 1 (11%) | 6 (67%) | 1 (11%) | 1 (11%) |
|  | Metastatic | 22 (85%) | 4 (15%) | 0 | 0 |
|  |  |  |  |  |  |
| Dysphagia | Total | 31 (89%) | 2 (8%) | 1 (3%) | 0 |
|  | Locoregional | 6 (67%) | 2 (22%) | 1 (11%) | 0 |
|  | Metastatic | 25 (100%) | 0 | 0 | 0 |
|  |  |  |  |  |  |
| Pain | Total | 27 (77%) | 7 (20%) | 0 | 1 (3%) |
|  | Locoregional | 7 (78%) | 1 (11%) | 0 | 1 (11%) |
|  | Metastatic | 20 (76%) | 6 (24%) | 0 | 0 |
|  |  |  |  |  |  |
| Hematologic (neutropenia) | Total | 23 (66%) | 3 (8%) | 7 (20%) | 2 (6%) |
|  | Locoregional | 6 (67%) | 1 (11%) | 1 (11%) | 1 (11%) |
|  | Metastatic | 17 (64%) | 2 (8%) | 6 (24%) | 1 (4%) |
